# Blockchain-enabled business model innovation in sector-coupled energy communities

**DOI:** 10.12688/openreseurope.20632.1

**Published:** 2025-06-19

**Authors:** Zia Lennard

**Affiliations:** 1 R2M Solution SAS, Roquefort-les-Pins, France

**Keywords:** energy communities, business model innovation, digital platforms, blockchain, automated remuneration, peer-to-peer trading, sector coupling, flexibility services, value proposition, decentralisation

## Abstract

This paper analyses business model innovation and digital platform alignment—including blockchain-enabled remuneration and automated settlement—in sector-coupled energy communities. Drawing on six diverse pilot deployments, we explore how modular digital platforms and blockchain technologies can support peer-to-peer trading, flexibility services, and integration across electricity, heating, and mobility sectors. The methodology combines systematic value proposition mapping with technical and market analysis to assess real-world user needs, operational challenges, and regulatory barriers. Findings show that blockchain and digitalisation can improve transparency and trust in community energy markets, but effective scaling requires regulatory adaptation, flexible business models, and sustained user engagement. The results provide actionable insights for both practitioners and policymakers aiming to advance digital innovation and business model alignment in the evolving landscape of energy communities.

## Introduction

The rapid transformation of Europe’s energy landscape is placing energy communities at the forefront of efforts to achieve decarbonisation, citizen participation, and local value creation. These communities, bringing together households, municipalities, and businesses, are uniquely positioned to harness distributed resources and support the integration of electricity, heating, mobility, and emerging technologies. However, the realisation of this potential depends not only on innovative business models, but also on the deployment of digital platforms that enable efficient, transparent, and automated coordination among diverse actors.

Recent developments in blockchain and digital platform technologies have opened new pathways for community-driven energy innovation. By enabling peer-to-peer trading, automated settlement, and secure value flows, these tools can help overcome long-standing barriers related to trust, data management, and multi-actor coordination. At the same time, the process of aligning business models with new digital capabilities is inherently iterative, shaped by local needs, technical constraints, and evolving regulatory frameworks.

This paper addresses these challenges by examining how business model innovation and blockchain-enabled digital platforms interact to support robust, sector-coupled energy communities. Drawing on lessons from six FEDECOM pilot deployments, we analyse the technical, organisational, and regulatory factors shaping the evolution of transparent, scalable community business models in practice. In doing so, we aim to provide a foundation for further research and practical action to advance the next generation of digital, sector-coupled energy communities.

## State of the art and conceptual foundations

### Business models for energy communities

The business model landscape for energy communities is rapidly evolving, with recent literature highlighting both the promise and fragmentation of current approaches. Energy communities are emerging as key players in the evolving energy landscape, fostering citizen participation and driving the transition towards sustainable energy systems. Business models are crucial for the success and sustainability of these communities, ensuring that benefits and costs are distributed equitably and that local knowledge is leveraged. The rise of peer-to-peer (P2P), community self-consumption, and transactive energy models reflects new business model configurations for local energy trading among various stakeholders.

### Blockchain technology in sector-coupled energy communities

Blockchain technology is increasingly recognized for its potential to revolutionize the energy sector by enabling new business models, fostering transparency, and supporting innovation ^1– 3^. Its core attributes—decentralization, immutability, and automation through smart contracts—make it particularly well suited for P2P energy trading, traceable transactions, and the integration of sector-coupled services such as electricity, heating, cooling, and mobility ^4– 7^. Blockchain supports local energy markets, enables direct trading among prosumers, and underpins new business models for energy communities ^4, 6^.

Several systematic reviews provide overviews of blockchain’s application in energy, emphasizing both the technology’s promise and its current limits ^1, 5, 8^. Case studies and conceptual frameworks further highlight the ways blockchain can reconfigure transaction structures, governance, and value flows ^4, 9, 10^. Blockchain approaches are viewed as highly relevant to P2P energy trading and transactive energy models, where trust, decentralised verification, and automated remuneration are central for scalable, inclusive participation ^11, 12^.

However, there are also concerns that, without careful design, such models may risk perpetuating existing inequalities. Reis *et al*. (2021) offer a comprehensive review of energy community business models, identifying a diversity of arrangements but noting a lack of systematisation and maturity across the sector ^13^. Several recent systematic reviews have mapped the complexities and diversity of energy community business models, focusing on P2P trading, sector-coupling, and the microeconomic dynamics among actors ^14– 19^. These studies highlight that while macroeconomic and policy frameworks are well explored, there is a need for more research on micro-level incentives, revenue streams, and mechanisms for sustained citizen engagement.

Maruf *et al*. (2024) similarly stress that while sector-coupled and renewable-based energy communities can deliver substantial environmental and economic benefits, practical deployment is hindered by technology integration, regulatory uncertainty, and limited business case development ^20^. Recent comparative reviews further underscore the diversity of energy community business models and the evolution of arrangements across the EU, highlighting both the proliferation of collective self-consumption, peer-to-peer trading, and aggregation schemes, and the variety of local adaptations ^21– 23^.

Regulatory and operational challenges are well documented. Lowitzsch *et al*. (2020) and Inês *et al*. (2020) point to the heterogeneity of governance models and the slow pace of legal harmonisation under EU frameworks such as RED II ^24, 25^. Ramsebner *et al*. (2021) and ETIP SNET (2021) highlight the complexity of sector coupling—integrating electricity, heating, mobility, and hydrogen systems—while stressing the need for holistic “system-of-systems” approaches that go beyond single-technology pilots ^26, 27^. Efkarpidis *et al*. (2022) and the EN-TRACK project (2019) identify gaps in KPI definition and validation ^28, 29^, while Mohammadi (2023) and Košnjek *et al*. (2025) highlight the broad range of externalities, actor diversity, and business model risk factors that must be considered for successful replication of energy community initiatives ^30, 31^. Community-ownership and governance innovations were covered by Universal Smart Energy Framework (USEF) in 2019 and International Renewable Energy Agency (IRENA) in 2020, who emphasised the importance of local participation, risk sharing, and flexibility as central to robust value proposition development ^32, 33^.

Digitalisation and platformisation are increasingly seen as necessary for energy community scale-up, particularly through enabling value co-creation, automated operations, and performance-based remuneration. However, recent project evidence notes persistent pain points, including fragmented asset management, slow digital uptake, lack of trusted data-sharing frameworks, and limited options for transparent revenue distribution ^34^. The FEDECOM value proposition analysis reveals that user archetypes—ranging from district system coordinators to e-mobility managers—face unique jobs, pains, and gains that are not fully addressed by legacy business models or generic digital solutions. The FLEXCoop project in 2020 demonstrated how digital platforms and marketplaces can facilitate local flexibility trading, introduce new contractual and risk-sharing mechanisms, and deliver additional value to both prosumers and aggregators ^35^.

Against this backdrop, blockchain and automated remuneration mechanisms have gained significant research attention as potential enablers of trust, transparency, and scalable value flows within local energy markets ^36, 37^. In the context of energy communities, blockchain can automate P2P transactions, enforce settlement logic based on KPIs, and facilitate multi-actor coordination. Yet, most academic and applied projects remain at proof-of-concept stage, with few documented cases of operational integration and business model impact at scale. Nonetheless, barriers to scale—including interoperability, regulatory fragmentation, and clarity of value stream allocation—remain, as consistently highlighted in recent academic and industry studies ^3, 22, 35^.

### Challenges, barriers, and research directions

Despite its promise, blockchain adoption in sector-coupled energy communities faces substantial challenges. Regulatory uncertainty, technological immaturity, scalability concerns, and data privacy are well documented as primary barriers ^38– 40^. Institutional inertia and limited awareness can slow adoption, while the need for interoperability and standardization remains acute ^4, 10^.

Still, the literature highlights significant benefits: increased efficiency through automation ^41^, sustainability via local renewables ^42^, improved resilience ^43^, and greater consumer empowerment ^4^. Future research should focus on scalable and secure platform design, business model innovation, socio-economic assessment, and progress toward technical interoperability ^6, 44^.

The present study builds on these insights, combining a systematic value proposition mapping across six energy community use cases with technical and market analysis from public FEDECOM deliverables. The goal is to demonstrate how digital business model innovation—integrating blockchain-based platforms and automated remuneration—can address real-world user needs, overcome regulatory and operational barriers, and create replicable value propositions for sector-coupled energy communities.

## Methodology and analytical approach

This research applies a multi-stage, mixed-methods approach to synthesise current business model innovation and value proposition development for energy communities, drawing on both peer-reviewed literature and recent European project experience. The analysis focuses on six representative energy community use cases from the FEDECOM project, selected to capture a diversity of market actors, technological contexts, and sector-coupling opportunities.

First, a qualitative review of academic and grey literature was performed to identify business model archetypes, value creation mechanisms, and recurring challenges in the evolution of energy communities ^14, 21, 22, 23, 12, 45, 11^. Special attention was given to systematic reviews and EU project reports that examine both macro- and micro-economic factors, with a particular focus on P2P, collective self-consumption, and digitally enabled business models ^15– 19^.

Personas and value proposition maps for the FEDECOM use cases were developed through project workshops, and analysis of deliverables. The Value Proposition Canvas framework ^46^ was used to structure and visualise the relationships between user jobs, pains, and gains, and the proposed products, services, pain relievers, and gain creators. The same structured approach was applied across all six use cases, facilitating a consistent basis for comparison and enabling iterative refinement as stakeholder feedback was incorporated.

For each persona, business model requirements were mapped to FEDECOM’s technical features—such as blockchain-enabled trading, automated remuneration, transparent transaction logs, and secure data exchange—by cross-referencing project deliverables and platform documentation with user needs identified in the value mapping. Iterative co-creation sessions with technical and market experts ensured alignment between the business model logic and the digital platform design. These sessions included regular feedback cycles during pilot deployment, enabling rapid identification and implementation of necessary adjustments in both business model logic and technical platform features.

Finally, FEDECOM pilot deployment and implementation reports were reviewed to evaluate the potential operationalisation of business models and the ability of digital features (e.g., smart contracts, KPI-based settlement) to address practical barriers and stakeholder expectations. This approach ensures that the methodology is transparent and reproducible, providing a template for comparable studies in future energy community projects. Where available, user feedback and observed market outcomes were synthesised to draw lessons on business model robustness, scalability, and transferability. This methodological integration enables a holistic assessment of how current theory and FEDECOM technical innovations interact to shape the next generation of energy community business models.

## FEDECOM business model innovation and platform alignment

The FEDECOM pilots provide an evolving perspective on how flexible, interoperable, and user-driven energy communities can be implemented in diverse real-world contexts. Rather than a finished product, FEDECOM’s approach to business model innovation and digital platform alignment is an ongoing, iterative process shaped by feedback from demonstration sites, technical development cycles, and continued stakeholder engagement.

**Figure 1.**
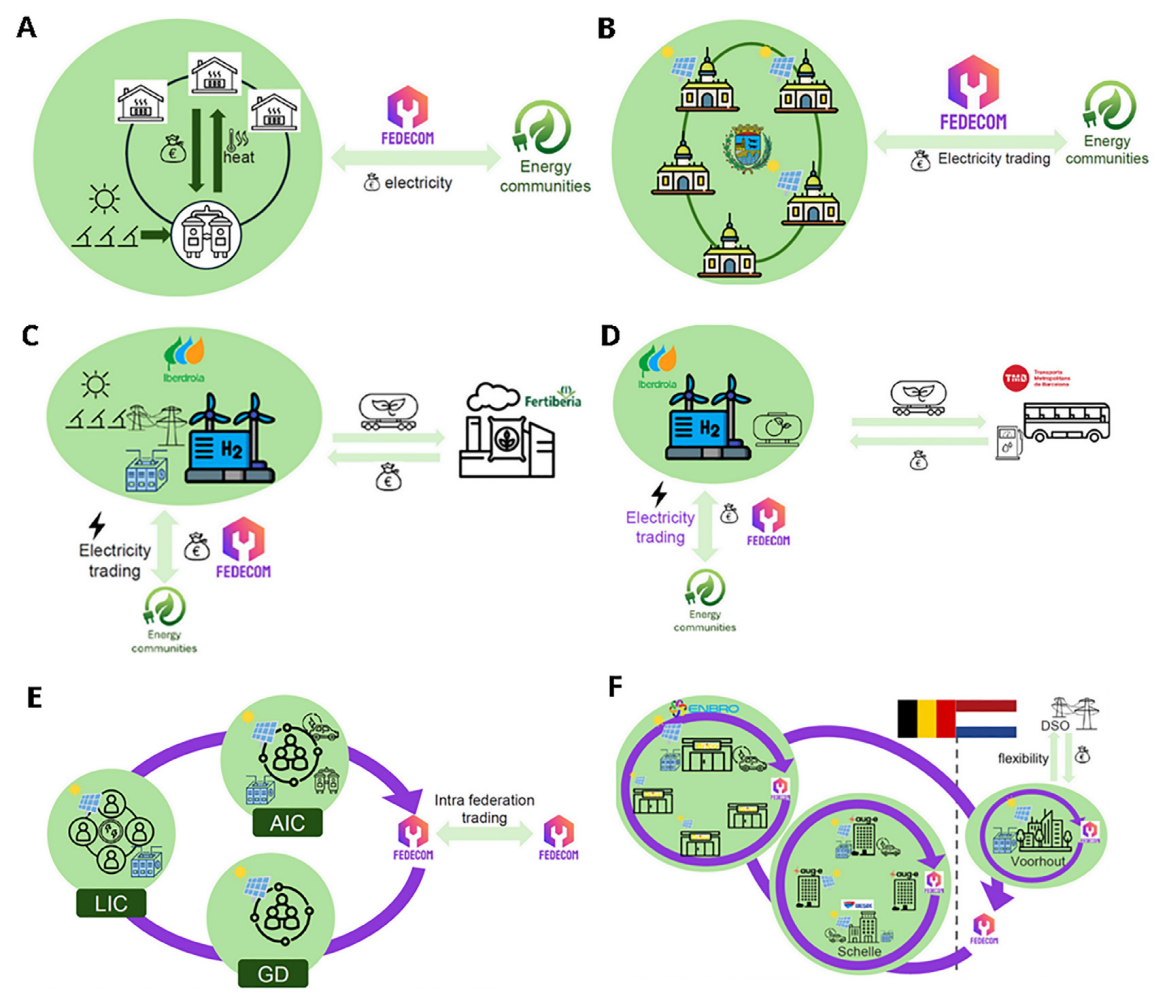
FEDECOM pilot business models and value propositions, including asset upgrades and main benefits, adapted from project factsheets (2024). ( **A**) UC1: Spain – Ur Beroa Residential Community. ( **B**) UC2: Spain – Bilbao City Hall. ( **C**) UC3: Spain – Puertollano Green Hydrogen Plant. ( **D**) UC4: Spain – TMB Hydrogen Station. ( **E**) UC5: Switzerland – Hydropower Federation. ( **F**) UC6: Benelux – Cross-country E-mobility.

Each pilot use case illustrates the challenges and opportunities inherent in adapting digital platform features and business model logic to local requirements, legacy constraints, and emerging value opportunities. The diversity of these approaches is visualised in Figure 1, which summarises the main business model configurations and value propositions across all six demonstration sites. For instance, in the Ur Beroa residential community in Spain ( Figure 1A), the integration of 37 kWp of new photovoltaics and 60 kWh of battery storage, together with improved cascading controls and the testing of phase change thermal storage, has enabled Ur Beroa’s operator to optimise a 50-dwelling district heating network that was previously reliant on a 1.2 MW CHP engine and 6.8 MW of natural gas boilers. This ongoing process has delivered higher self-consumption, improved cost savings, and prepared the community for local trading and sector coupling. The Bilbao City Hall case ( Figure 1B) shows how municipal portfolio management and renewable integration are being dynamically coordinated across six public buildings and two PV sites (totaling 1044 kWp of generation and 127 kWh of storage). FEDECOM enabled automated monitoring, real-time data integration, and improved reporting, facilitating both internal optimisation and readiness for future local energy trading.

Similarly, the Puertollano Green Hydrogen plant ( Figure 1C) demonstrates the integration of a 100 MW green hydrogen plant, 100 MW of PV, and a 20 MWh battery, with FEDECOM enabling optimal dispatch between electricity and hydrogen production. The platform supports green credential tracking and paves the way for multi-market participation, though further work on regulatory adaptation and commercial integration is ongoing. The TMB hydrogen station pilot ( Figure 1D) highlights the acceleration of energy trading for public transport hydrogen infrastructure, with the platform supporting four new dispensers, expanded high-pressure storage, and secure links to a green hydrogen plant and PV. While FEDECOM has prepared TMB for broader market integration, further expansion and regulatory adaptation are still being addressed. In Switzerland, the hydropower federation pilot ( Figure 1E) coordinated three communities with over 250 kWp of PV, new district batteries, and legacy hydro assets. FEDECOM enabled optimisation of self-consumption, ancillary service provision, and transparency in asset coordination, but also revealed the need for ongoing user training and iterative business model adaptation. The Benelux cross-country e-mobility pilot ( Figure 1F) linked PV-powered EV charging sites across Belgium and the Netherlands, with integration of battery storage, smart charging, and dynamic control to enable local and cross-border electricity, flexibility, and storage trading. FEDECOM’s modular platform provided technical feasibility, while highlighting the need for new special purpose vehicles and regulatory alignment for full commercial rollout.

Across all use cases, value proposition mapping and business model adaptation remain iterative, shaped by real-time feedback and local user priorities. The platform’s ability to support transparent accounting, smart contract-based settlement, and flexible user roles has been instrumental in enabling these adaptations, even as regulatory fit and full integration with legacy systems continue to present challenges.

The diversity of FEDECOM’s pilot sites is reflected not only in their business models but also in the specific platform functionalities and market roles addressed by the project. Table 1 summarises the key FEDECOM market offers, the main stakeholder groups targeted, and the use cases where each offer was implemented or tested.

**Table 1.** Mapping of FEDECOM platform market offers to stakeholder groups and use cases, based on pilot factsheets (2024).

FEDECOM offers	To	Use cases
Local Electricity Market	Energy communities Real estate owners	UC2, UC5, UC6
Local Flexibility Market	Energy communities Real estate owners	UC2, UC5, UC6
Inter-energy community energy trading	Cluster of energy communities Large industrial energy consumers	UC1, UC2, UC3, UC4, UC5, UC6
Electricity storage trading	Energy communities DHC network operators Real estate owners Electric vehicle fleet owners	UC1, UC3, UC5, UC6
P2X trading	Energy communities Hydrogen manufacturers DHC network operators Hydropower companies	UC1, UC3, UC4, UC6

As shown in Table 1, the platform enables local electricity and flexibility markets, supports inter-community energy trading, electricity storage trading, and P2X (power-to-x, e.g., hydrogen) trading. Each offer corresponds to different user groups—ranging from real estate owners, district heating (DHC) operators, and large industrial consumers to clusters of renewable energy communities and EV fleet operators. This mapping highlights the flexibility of the platform to accommodate a variety of trading and value creation logics, tailored to the operational and market realities of each pilot. For example, in Bilbao, real-time monitoring, automated control, and enhanced transparency provided clear benefits to municipal managers, while in Ur Beroa, cost savings and increased resilience were observed as key outcomes for residents. Across all pilots, transparent accounting, automated settlement, and flexibility management were highly valued by both technical operators and end users, though full integration remains a work in progress in several sites.

The mapping process also highlighted where blockchain-specific features—such as smart contract settlement and auditability—were critical to value proposition delivery, particularly in multi-actor, cross-community, or sector-coupled use cases.

**Figure 2.**
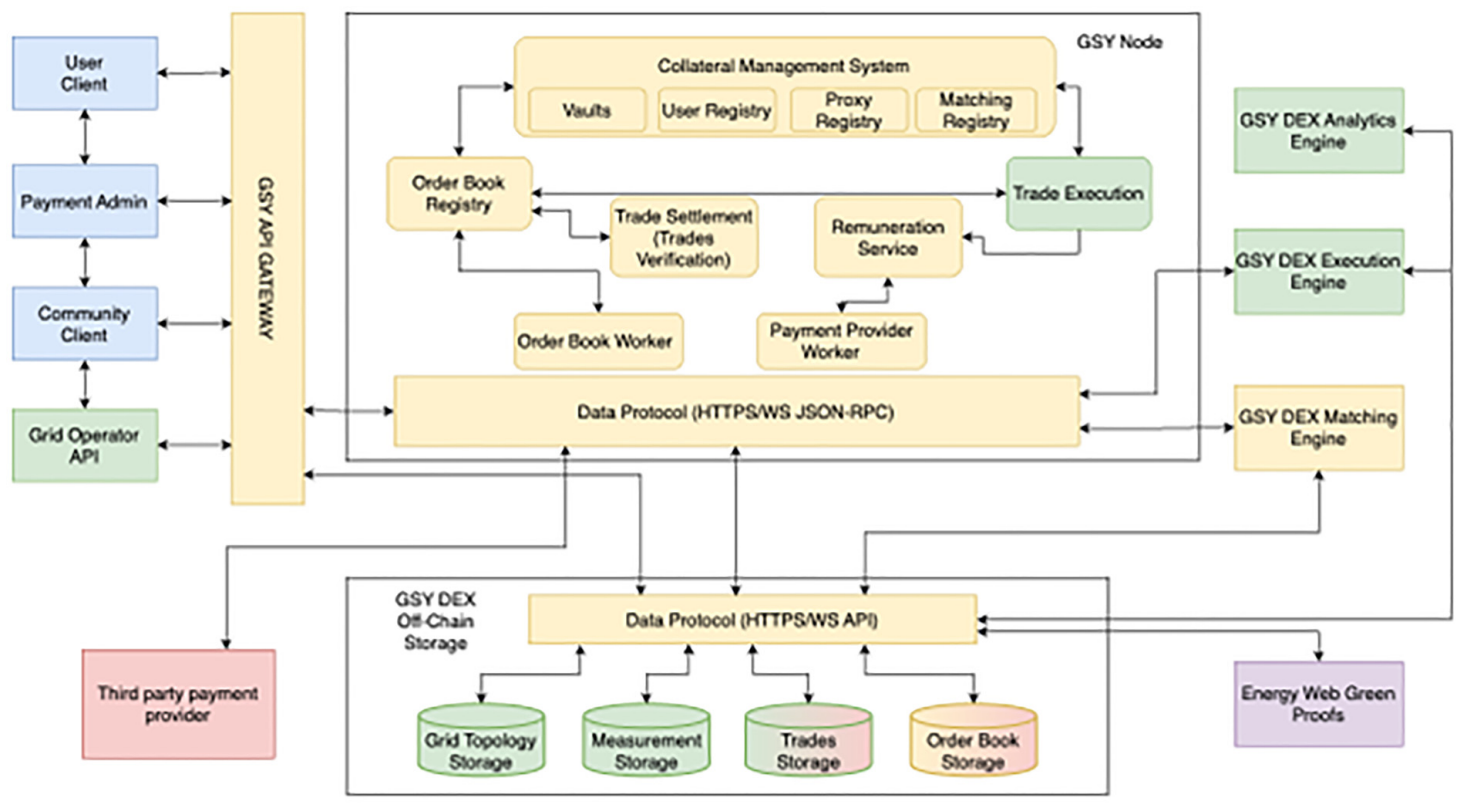
System architecture of the FEDECOM GSY DEX platform, showing blockchain-based market, remuneration, analytics, and payment integration. Adapted from FEDECOM D6.1.

FEDECOM’s technical backbone, the Grid Singularity (GSY) Distributed Energy Exchange (DEX) platform, is visualised in Figure 2. This modular blockchain-based architecture underpins secure trading, performance verification, and automated remuneration for all pilots. The architecture enables order matching, trade execution, KPI-driven settlement, and integration with external analytics or payment providers. Importantly, the platform’s flexibility has allowed project partners to rapidly test new incentive structures, user permission schemes, and market logic configurations in response to pilot feedback.

As pilots progress, continuous improvement is guided by lessons learned from each demonstration site, technical user feedback, and evolving regulatory environments. For example, the BeNeLux Cross-country e-mobility pilot (Use Case 6) underwent substantial adaptation during the project, and the results described reflect both implemented features and ongoing work. The factsheets highlight ongoing challenges: for example, user training and legacy integration in the Swiss federation, regulatory negotiation for cross-border trading in BeNeLux, and iterative technical adaptation in the hydrogen and municipal pilots. The FEDECOM experience demonstrates that while robust, modular digital platforms and business model innovation are essential enablers, true alignment is achieved through ongoing co-design, flexible adaptation, and sustained stakeholder partnership.

## Discussion and implications

The FEDECOM pilot experiences reinforce that business model innovation and digital platform alignment for energy communities must be understood as adaptive, iterative, and context-dependent processes. The diversity of outcomes across pilots—from Spanish district heating to Swiss hydropower to cross-border e-mobility—demonstrates that both technical and organisational pathways are shaped by legacy infrastructure, regulatory environments, and evolving user needs.

One central lesson is that co-design between platform developers, technical partners, and local actors is essential. In several pilots, such as Ur Beroa and Bilbao City Hall, transparent real-time monitoring and automated settlement created immediate value, but also surfaced new requirements for user support and integration with existing systems. Where technical upgrades enabled new flexibility, storage, or trading capabilities, business models were adjusted in parallel—a process often requiring multiple iterations of user training and feedback.

The pilot factsheets highlight that technological enablement alone does not guarantee operational or financial success. For instance, while the FEDECOM platform allowed the Puertollano Green Hydrogen Plant and TMB Hydrogen Station to trial advanced energy management and market participation, the actual commercial impact is still dependent on regulatory approval and business model maturity. Similarly, in the BeNeLux e-mobility federation, technical feasibility of cross-border energy trading was demonstrated, but full implementation remains subject to legal, market, and organisational challenges.

Replication and scalability emerged as both opportunity and challenge. The Swiss federation’s positive experience with flexibility sharing and transparent data analytics points to pathways for replication elsewhere but also underscores the ongoing need for stakeholder engagement and adaptation to local context. Across all pilots, feedback repeatedly emphasised that the platform’s value was maximised when customisation—of business logic, user interface, and reporting—was prioritised.

A persistent barrier is regulatory and market alignment. Even with a flexible, modular platform, pilots encountered shifting requirements, uncertain remuneration pathways, and sometimes lengthy approval processes. These findings confirm that digital platform innovation must proceed in lockstep with policy advocacy and regulatory dialogue, not as an isolated technical exercise.

The FEDECOM experience also points to several forward-looking implications. First, open, modular platforms are crucial for enabling communities to experiment with new roles and business models as market rules evolve. Second, meaningful and ongoing stakeholder co-creation—including feedback loops, capacity building, and transparent communication—are prerequisites for sustainable deployment. Third, as community energy grows in scale and complexity, continuous improvement in both platform technology and business models is necessary, informed by real-world data and emerging best practices. Future work should also benchmark the scalability, energy use, and user acceptance of blockchain-based platforms versus centralised alternatives in operational settings.

In summary, FEDECOM’s pilot phase demonstrates that digital platform and business model innovation for energy communities is an ongoing, collaborative process. The platform’s modularity and flexibility allowed diverse communities to test, adapt, and advance new solutions, while also revealing the central importance of local engagement and regulatory fit. These insights should inform not only the next phase of FEDECOM but also the wider community energy sector as it moves toward a more decentralised, digital, and participatory future.

## Conclusions and future directions

This study demonstrates that innovation in energy community business models and digital platform design—particularly those leveraging blockchain-enabled architectures—is both essential and inherently iterative. The FEDECOM pilots across Spain, Switzerland, and Benelux show that a modular, blockchain-based platform can support transparency, automation, and secure multi-actor value flows for a wide range of sector-coupled community models. Real-world deployments confirmed that technical upgrades such as smart contract-based settlement, auditability, and automated remuneration, when aligned with clear business models and local needs, enable new forms of trust, participation, and operational efficiency.

However, the experience also underscores that digital platforms and blockchain are not one-size-fits-all solutions. Success depends on careful co-design with users, robust technical integration, and ongoing adaptation to regulatory and market changes. Achieving robust and replicable energy community solutions requires a combination of digital innovation, flexible business model design, and continuous engagement with stakeholders at all levels.

Looking ahead, future work should further benchmark the scalability, energy efficiency, and user acceptance of blockchain-based versus centralised solutions in operational community settings. Continued research is also needed to advance interoperability, support sector-coupling, and enable integration with third-party and external market platforms. Most critically, policy and regulatory frameworks must keep pace with digital innovation, ensuring that communities can benefit fully from the transparency, automation, and resilience that these platforms offer.

In summary, the FEDECOM pilots confirm that digitally enabled, blockchain-based platforms—when matched to context-specific business models and implemented through close collaboration—offer a viable path forward for sector-coupled, community-driven energy transitions across Europe.

## Ethics and consent

No ethics and consent were required.

## Disclaimer

The views expressed in this article are those of the author. Publication in Open Research Europe does not imply endorsement of the European Commission.

## Data Availability

No data are associated with this article.
