# Supplementary material for: Blockchain-enabled business model innovation in sector-coupled energy communities
Source: Open Res Eur. 2025 Jun 19;5:168. [Version 1] doi: 10.12688/openreseurope.20632.1 (PMC12640504; doi:10.12688/openreseurope.20632.1)
Supplement: Peer review history

# Reviewer report: Reviewer response for version 1

**DOI:** 10.21956/openreseurope.22322.r60058

**Published:** 2025-10-03

**Authors:** Assunta Di Vaio

**Affiliations:** 1 University of Naples “Parthenope”, Naples, Italy

This Open Letter titled Blockchain-enabled business model innovation in sector-coupled energy communities explores the linkages between business model innovation and blockchain-enabled digital platforms in the context of sector-coupled energy communities. The topic is highly relevant, and the empirical material provided by the FEDECOM pilot projects offers useful insights that go beyond conceptual speculation. The document clearly has the potential to make a contribution both to academic debate and to practice. However, several issues require further refinement before this document can reach the level of rigour. My overall recommendation is for minor revisions, although the issues I outline are essential for strengthening this Open Letter titled Blockchain-enabled business model innovation in sector-coupled energy communities.

 The abstract is clear and well organised, summarising the scope of the study and the key results. Nonetheless, it remains at a mainly descriptive level. The author should use this section to signal more explicitly their theoretical contribution, rather than focusing almost exclusively on pilot results. Highlighting how the study advances academic debates on business model innovation and digital transformation would make the abstract more balanced and impactful.

 The introduction successfully positions energy communities within the broader European decarbonization agenda, emphasising their role in fostering citizen participation and sector integration. Yet, the research gap is somewhat implicit. The abundance of existing conceptual and proof-of-concept studies contrasts with the limited number of empirical analyses of real-world business models integrated with blockchain-enabled platforms. This tension should be highlighted more directly to justify the necessity of the study. Likewise, the research question is discernible but never explicitly stated. Formulating it clearly and concisely would help all readers understand precisely how the paper aims to contribute to the literature.

 The literature review demonstrates breadth but not always depth. It presents a wide range of sources on business models, blockchain applications, and regulatory barriers, but the narrative occasionally becomes fragmented, listing findings without synthesising them into a coherent framework. The authors could improve this section by structuring it more clearly around categories such as business model archetypes, digitalisation and trust, and governance and regulation. Moreover, the review would benefit from engagement with broader strands of research beyond the immediate energy community field. For example, work published in journals such as the *Journal of Enterprise Information Management* has examined how digitalisation and accountability mechanisms reshape transparency and value flows, which could reinforce the discussion of blockchain’s role in enabling trust. Studies in *Production Planning & Control* have analysed inter-organisational relationships in logistics and port operations, offering useful parallels for understanding platformisation and multi-actor coordination in energy systems. Research in *Scientific Reports* has recently investigated the interaction between environmental uncertainty, digital technologies, and corporate green behaviour, which could provide a richer foundation for discussing adaptive and flexible business models under regulatory and market uncertainty. Finally, systematic literature reviews in the *Journal of Business Research* have explored the role of digital innovation in knowledge management systems, offering a framework for interpreting blockchain not only as a technical infrastructure but also as a mechanism for distributed knowledge creation and sharing. You can also find several papers on business models and technological innovation in the Journal of Business Research and the Journal of Cleaner Production. Integrating these streams would allow the manuscript to connect with wider theoretical debates and situate its contribution more firmly in the literature.

 The methodology section is promising, as it employs a mixed-methods approach that combines literature review, value proposition mapping, and analysis of six pilot cases. The use of the Value Proposition Canvas is appropriate for capturing user needs and linking them with digital platform features. However, the justification for this methodological choice remains underdeveloped. The authors should connect their approach to existing studies that have applied similar frameworks in contexts of digital innovation, business model design, or knowledge management. Without this link, the method risks appearing arbitrary or too closely tied to the specific project. The section would also be strengthened by clarifying the nature of the data sources, the role of stakeholders, and the way feedback loops were managed across pilot sites. This would increase the transparency and reproducibility of the analysis.

 The results section provides a rich and detailed description of each pilot case, outlining the technological features tested and the organisational arrangements explored. While this descriptive detail is valuable, the analysis does not always rise above the level of case-by-case reporting. The paper would benefit from more explicit comparative analysis across cases, drawing out the common barriers, recurring challenges, and transferable solutions. A stronger cross-case synthesis would allow the results to move from descriptive accounts toward more generalisable insights.

 The discussion section identifies important results, such as the iterative nature of business model adaptation and the need for co-design with stakeholders. These are indeed key lessons, but the discussion currently reads more as an extension of the results rather than a critical engagement with the literature. The author should highlight more clearly how the results confirm, extend, or challenge prior studies, particularly in relation to theories of digital transformation, business model innovation, and knowledge management. For example, the claim that blockchain enhances trust and transparency could be situated in the literature on accountability and digitalization, while the observation that business models evolve iteratively could be linked to open innovation or dynamic capabilities frameworks. Establishing these theoretical connections would greatly enhance the scholarly significance of the discussion.

 The implications of this study are promising but need to be elaborated. On the theoretical side, the authors should clarify how the findings contribute to ongoing debates about digital innovation and business models in decentralised systems. At present, the theoretical implications are noted but remain too general. On the practical side, the recommendations are useful but presented at a high level of abstraction. Rather than simply calling for regulatory adaptation or user engagement, the paper should provide more concrete guidance that distinguishes between the needs of policymakers, community operators, and technology providers. Offering stakeholder-specific recommendations would make the paper’s practical relevance clearer and more actionable.

 The conclusion is concise and provides a good summary of the study. However, it could do more to underline the dual contribution to theory and practice. The authors should restate explicitly how the empirical evidence advances theoretical understanding of business model innovation and digitalisation, while also drawing out more concrete lessons for practitioners. Future research directions should not be limited to technical benchmarking but should also include governance, accountability, and the long-term sustainability of digital platforms in energy communities.

 In summary, this ‘Open Letter’ is based on valuable empirical material and addresses an important question for the energy transition. It demonstrates convincingly that business model innovation and blockchain-enabled digital platforms can support sector-coupled energy communities, but it does not yet fully capitalise on its potential to contribute to the academic literature. To achieve this, the paper should make the research gap and question explicit, strengthen the literature review with broader and better synthesised sources, justify the methodological choices with reference to similar studies, provide deeper cross-case analysis in the results, connect the discussion more explicitly to theoretical debates, and expand both theoretical and practical implications. If these revisions are undertaken, the manuscript will stand as a meaningful contribution to scholarship on energy communities, digital transformation, and business model innovation, while also offering actionable insights for practitioners and policymakers engaged in the transition toward decentralised and sustainable energy systems.

 I have observed that this study has been categorized as an Open Letter. In my view, this classification does not fully reflect the nature of the work. Given its empirical foundation, methodological approach, and the evidence presented through pilot cases, the manuscript would be more appropriately classified as an empirical research article. Reconsidering the categorization would ensure that the paper is properly positioned within the journal’s publication typologies and that its contribution is evaluated accordingly.

Where applicable, are recommendations and next steps explained clearly for others to follow? (Please consider whether others in the research community would be able to implement guidelines or recommendations and/or constructively engage in the debate)

Not applicable

Does the article adequately reference differing views and opinions?

Partly

Are all factual statements correct, and are statements and arguments made adequately supported by citations?

Partly

Is the rationale for the Open Letter provided in sufficient detail? (Please consider whether existing challenges in the field are outlined clearly and whether the purpose of the letter is explained)

Partly

Is the Open Letter written in accessible language? (Please consider whether all subject-specific terms, concepts and abbreviations are explained)

Partly

Reviewer Expertise:

Sustainability Accounting and Management

I confirm that I have read this submission and believe that I have an appropriate level of expertise to confirm that it is of an acceptable scientific standard, however I have significant reservations, as outlined above.

# Response

**Published:** 2025-10-06

**Authors:** Zia Lennard

**Affiliations:** R2M Solution, France

I am sincerely grateful for the reviewer’s thoughtful and encouraging feedback. The detailed comments have helped me strengthen the analytical rigor and scholarly positioning of the manuscript while remaining faithful to the concise format required by *Open Research Europe*’s Open Letter classification. Below I address each point in turn.

**1. Abstract and theoretical contribution**

 The abstract has been revised to better communicate the study’s theoretical contribution. I added a sentence explicitly highlighting how the analysis advances understanding of digitalisation and distributed governance in energy communities by linking iterative co-design and blockchain-based verification to broader theories of digital transformation and value creation.

**2. Research gap and explicit research question**

 The introduction was expanded to make the research gap clearer. I added two sentences explicitly contrasting the abundance of conceptual studies with the lack of empirical evidence on real-world, blockchain-integrated energy community models. Immediately following this clarification, I inserted a concise research question that directly frames the paper’s analytical focus.

**3. Literature review scope and structure**

 I fully acknowledge the reviewer’s valuable suggestions regarding the breadth and synthesis of the literature review. However, given *Open Research Europe*’s revision procedure for Open Letters—which does not allow major structural reorganisation or insertion of new, external academic material beyond the approved manuscript scope—it was not possible to extend the review to include additional academic domains such as enterprise information management, logistics, or knowledge management. Instead, I improved the internal coherence of the existing section by clarifying transitions between paragraphs and reinforcing the synthesis of cited studies around three conceptual strands: business model innovation, digitalisation and trust, and governance and regulation. This approach maintains readability while aligning with the reviewer’s suggestion for a clearer conceptual structure.

**4. Methodology justification and reproducibility**

 The methodology section has been expanded to justify the multi-stage, mixed-methods approach in relation to established frameworks for business model design and digital innovation. I clarified how the Value Proposition Canvas was operationalised through iterative co-creation and feedback loops across the six pilot sites, and added a short paragraph explaining how this ensures methodological transparency and reproducibility.

**5. Cross-case synthesis in the results**

 To strengthen analytical depth, I introduced a new synthesis paragraph following the pilot descriptions. This identifies recurring themes and transferable innovations across the six cases, highlighting shared patterns in stakeholder engagement, platform integration, and flexibility monetisation.

**6. Discussion and theoretical engagement**

 I reworked the discussion section to better connect empirical findings to theoretical debates. The text now explicitly references how the results confirm or extend existing perspectives on open innovation, digital accountability, and iterative business model adaptation. This reframing underscores the paper’s dual contribution to both theory and practice.

**7. Practical implications and stakeholder-specific recommendations**

 The implications section was expanded to distinguish between the needs of policymakers, community operators, and technology providers. Each group now has tailored recommendations, improving the paper’s practical relevance and direct applicability.

**8. Strengthening the conclusion**

 I revised the conclusion to restate the dual contribution to theory and practice. The final paragraph now highlights how empirical insights from the pilots enhance understanding of digitalisation in decentralised systems while also offering practical lessons for implementing scalable, transparent, and resilient energy communities. I also added a sentence identifying governance, accountability, and long-term platform sustainability as key directions for future research.

**9. On classification as an Open Letter**

 I appreciate the reviewer’s view that the paper might better fit the “empirical research article” category. However, since the initial submission was accepted under the Open Letter format, reclassification would require editorial approval. I have nevertheless ensured that the revised version meets the rigour expected of an empirical contribution while maintaining the format conventions of the Open Letter type.   I believe these targeted revisions fully address the reviewer’s recommendations and enhance both the scholarly depth and practical clarity of the paper while respecting *Open Research Europe*’s formal revision process.

# Reviewer report: Reviewer response for version 1

**DOI:** 10.21956/openreseurope.22322.r60064

**Published:** 2025-09-17

**Authors:** Ángel Paredes

**Affiliations:** 1 Universidad de Málaga, Málaga, Spain; 2 Universidad de Sevilla Escuela Tecnica Superior de Ingenieria de Sevilla, Seville, Andalusia, Spain

This open letter analyses the integration of blockchain-enabled digital platforms with business model innovation across six diverse, sector-coupled energy community pilots within the FEDECOM project. The topic is highly timely and addresses a critical research gap at the intersection of digitalization, decentralization, and energy system integration. However, while the letter is well-structured and its objectives are clear, its potential impact is hampered by a descriptive rather than analytical approach, a lack of measurable results, and missed opportunities to connect findings to broader industry initiatives. The following remarks are offered to strengthen the manuscript:

 1. The methodology, described as a "multi-stage, mixed-methods approach," remains vague. A more rigorous justification is needed, clarifying how the synthesis of workshop outputs and literature review constitutes a novel methodological contribution compared to established practices in business model analysis for energy communities. Furthermore, the analysis would be significantly enriched by contextualizing its findings within existing standardization efforts, notably the BRIDGE Business Models Working Group initiative, to clarify how FEDECOM's approach aligns with or diverges from current European best practices.

 2. The core goal of demonstrating "replicable value propositions" is not fully met. The letter would be substantially strengthened by moving beyond case-specific descriptions to synthesize a generalized, demo-wide value proposition framework. A powerful way to achieve this would be to present a Lean Model Canvas that conceptualizes the commonalities of the blockchain-enabled business model across all pilots, explicitly mapping its contributions and disruptions to the traditional energy value chain.

 3. Figure 1, in its current form, functions merely as a summary of pilot assets and lacks analytical value. This figure should be revised or supplemented to visually articulate the innovation in each business model and value propositions for each case.

 4. Section "FEDECOM business model innovation and platform alignment" suffers from a "specific-to-general" with unclear innovations. It should be reorganized to first present the overarching business model logic and architectural framework of the FEDECOM platform (the general), before then detailing how each pilot instantiates and adapts this model (the specific). This would provide readers with a clear analytical framework before diving into the case details, making the business model innovations far more comprehensible.

 5. The discussions on regulatory barriers are not paired with sufficiently concrete or actionable recommendations. The letter must go beyond stating that "policy must keep pace" and provide specific, actionable and standardized proposals for regulators that would directly facilitate the adoption of these technologies.

 6. The design of smart contracts is the operational heart of the proposed blockchain-based business models, yet it is treated as a black box. The letter misses a critical opportunity to provide actionable insights for practitioners by discussing the essential clauses, logic, and dependencies required in these contracts to automate remuneration, manage flexibility, and enforce KPI-based settlements effectively.

 7. A significant omission is a discussion of the trade-offs involved in using blockchain. The letter states the benefits of transparency and automation but neglects the inherent disadvantages, such as the energy consumption of the consensus mechanism, transaction costs (gas fees), and computational complexity.

Where applicable, are recommendations and next steps explained clearly for others to follow? (Please consider whether others in the research community would be able to implement guidelines or recommendations and/or constructively engage in the debate)

Partly

Does the article adequately reference differing views and opinions?

Partly

Are all factual statements correct, and are statements and arguments made adequately supported by citations?

Partly

Is the rationale for the Open Letter provided in sufficient detail? (Please consider whether existing challenges in the field are outlined clearly and whether the purpose of the letter is explained)

Yes

Is the Open Letter written in accessible language? (Please consider whether all subject-specific terms, concepts and abbreviations are explained)

Partly

Reviewer Expertise:

Business models analysis, market participation strategies, artificial intelligence, DER management.

I confirm that I have read this submission and believe that I have an appropriate level of expertise to state that I do not consider it to be of an acceptable scientific standard, for reasons outlined above.

# Response

**Published:** 2025-10-06

**Authors:** Zia Lennard

**Affiliations:** R2M Solution, France

I sincerely thank you for your detailed and constructive feedback. The comments have been invaluable in strengthening the analytical depth and methodological clarity of this revised version. All seven points have been addressed as follows:

**1. Methodology clarification and alignment with standardisation efforts**

 I have substantially revised the methodology section to clarify the structure and novelty of the “multi-stage, mixed-methods” approach. The text now explicitly distinguishes between the qualitative (stakeholder workshops, value proposition mapping) and quantitative (KPI validation and comparative analysis) components. I also added a short paragraph linking the FEDECOM methodology to established European practices, referencing the alignment with ongoing work under the BRIDGE Business Models Working Group and other EU-funded initiatives. This demonstrates how the applied approach contributes to harmonising business model evaluation frameworks.

**2. Demo-wide synthesis of replicable value propositions**

 To address this comment, I added a new paragraph following the description of the iterative co-design process, synthesising cross-pilot findings into a generalised value proposition framework. The text highlights shared innovation patterns, recurring value creation mechanisms, and transferable design elements. While a Lean Model Canvas format was considered, the synthesis was presented in narrative form to remain consistent with ORE’s Open Letter format.

**3. Revision of Table 1 for analytical depth**

 I revised Table 1 (previously Figure 1) and its accompanying text to move beyond a descriptive listing of pilots. It now integrates analytical information regarding innovation focus, stakeholder relevance, and measurable impacts across pilots. The caption and surrounding paragraphs were rewritten to explain how these patterns illustrate the platform’s cross-sector scalability and interoperability.

**4. Reorganisation of “FEDECOM business model innovation and platform alignment”**

 This section has been restructured to follow a “general-to-specific” logic. It now begins with a description of the overarching business model architecture, followed by pilot-level adaptations. A transition sentence was inserted to signal this change to readers, improving coherence and analytical flow.

**5. Regulatory recommendations**

 I expanded the discussion of regulatory barriers to include specific recommendations. These include the need for harmonised energy-sharing frameworks, recognition of community aggregators as market participants, and simplified mechanisms for cross-border flexibility trading. This provides a more actionable basis for policymakers.

**6. Smart contract design**

 In Section 4, I added a new paragraph describing the essential logic underpinning smart contracts used for settlement and KPI-based remuneration. This includes clauses related to validation, collateral management, and flexibility activation, offering practical insights into how blockchain enables automated verification within decentralised energy markets.

**7. Discussion of blockchain trade-offs**

 I introduced a critical discussion of blockchain’s limitations in Section 5, addressing computational complexity, transaction costs, and energy consumption. The revised text explains how FEDECOM mitigates these trade-offs through a permissioned ledger and delegated validation, reducing the energy footprint while preserving transparency. Overall, these revisions make the paper more analytical, more transparent in its methods, and better aligned with European best practices for business model innovation and digitalisation in energy communities.

# Reviewer report: Reviewer response for version 1

**DOI:** 10.21956/openreseurope.22322.r55877

**Published:** 2025-07-09

**Authors:** Tiago Soares

**Affiliations:** 1 Center for Power and Energy Systems, INESC TEC, Porto, Portugal

This paper presents findings from the FEDECOM project, exploring how blockchain-enabled digital platforms can support business model innovation in sector-coupled energy communities. It is based on six pilot deployments across Europe and utilizes a structured methodology including value proposition mapping, stakeholder engagement, and analysis of technical and regulatory challenges. The paper argues that blockchain enhances transparency, trust, and automation, but must be paired with flexible, co-designed business models and ongoing regulatory alignment. The paper is interesting and well written; however, some points can be improved, namely:

1. The pilots descriptions are interesting, but there is a lack of KPIS proving the benefits of such an approach. Perhaps, some KPIS can be included in the analysis to understand at to what extent the developed approach improves cost savings.
2. The benefits of using blockchain are emphasized in the paper, but the main disadvantages like energy use, complexity and costs are not considered. Perhaps a critical discussion of the trade-off between using the blockchain in detriment of the centralized approaches can be addressed.
3. The pilots are interesting and different. However, the replicability and scalability of the platform to other levels a is not addressed. Perhaps it can be provided a list outlining prerequisite for replication of the platform.
4. The paper addresses the regulatory uncertainty as a key barrier but does not provide clear contextual regulatory examples and how specific changes could unlock the issue.
5. It would be good to have an idea of how blockchain impacts energy consumption in each of the pilots to add credibility to the adopted solution.

Where applicable, are recommendations and next steps explained clearly for others to follow? (Please consider whether others in the research community would be able to implement guidelines or recommendations and/or constructively engage in the debate)

Partly

Does the article adequately reference differing views and opinions?

Yes

Are all factual statements correct, and are statements and arguments made adequately supported by citations?

Yes

Is the rationale for the Open Letter provided in sufficient detail? (Please consider whether existing challenges in the field are outlined clearly and whether the purpose of the letter is explained)

Partly

Is the Open Letter written in accessible language? (Please consider whether all subject-specific terms, concepts and abbreviations are explained)

Yes

Reviewer Expertise:

Energy communities

I confirm that I have read this submission and believe that I have an appropriate level of expertise to confirm that it is of an acceptable scientific standard, however I have significant reservations, as outlined above.

# Response

**Published:** 2025-10-06

**Authors:** Zia Lennard

**Affiliations:** R2M Solution, France

I sincerely thank the reviewer for the careful reading and constructive comments. I am grateful for the positive assessment of the paper’s relevance, structure, and clarity, and have carefully addressed all points raised.

**1. Inclusion of KPIs to demonstrate benefits**

 I agree that the initial version did not sufficiently highlight quantifiable indicators. Section 4 has been expanded to include representative key performance indicators related to energy savings, flexibility activation, and remuneration accuracy. These examples illustrate how measurable validation results underpin the assessment of business model performance.

**2. Discussion of blockchain disadvantages and trade-offs**

 I have added a new paragraph in Section 5 discussing energy use, computational complexity, and transaction costs. This addition compares blockchain-based architectures with centralised approaches and clarifies how FEDECOM’s permissioned ledger design mitigates energy and latency challenges while maintaining transparency and auditability.

**3. Replicability and scalability prerequisites**

 To address this valuable comment, I added a paragraph outlining preconditions for replication, including interoperability, governance recognition, regulatory alignment, and local financial capacity. This section now also references the FEDECOM Replication Playbook and Self-Assessment Tool as resources supporting scalability across contexts.

**4. Regulatory examples and actionable proposals**

 I expanded the discussion on regulatory uncertainty to include concrete examples of permitting, perimeter definitions, and flexibility market access. The new text explains how harmonised recognition of energy communities as market participants could remove barriers to platform adoption.

**5. Blockchain energy consumption across pilots**

 I added a paragraph clarifying that FEDECOM’s platform uses a lightweight permissioned architecture with delegated validation and off-chain storage, which minimises energy use and computational demand. This contextualises blockchain efficiency within pilot operations and strengthens the credibility of the approach. I believe these revisions comprehensively address all points raised and significantly enhance the analytical depth and transparency of the paper.
